# Is the sitting position safe in posterior fossa surgery? An analysis of air embolism risk, complications, and surgical outcomes

**DOI:** 10.1007/s10143-026-04202-3

**Published:** 2026-03-31

**Authors:** Ali Nehir, Yiğit Aksoğan, Necati Üçler, Berna Kaya Ugur, Abidin Murat Geyik

**Affiliations:** 1 Department of Neurosurgery, Faculty of Medicine, Gaziantep University, Gaziantep, Turkey; 2 Department of Anesthesiology and Reanimation, Faculty of Medicine, Gaziantep University, Gaziantep, Turkey

**Keywords:** Posterior fossa surgery, Sitting position, Prone position, Air embolism risk, Complications, Surgical outcomes

## Abstract

In posterior fossa surgery, patient positioning (sitting, prone, or park-bench) requires a balance between surgical exposure, blood loss, and hemodynamic effects. Due to inter-center variability and case heterogeneity in the literature, no definitive superiority has been demonstrated. This study compared the effects of different positions on surgical outcomes and complications based on a single-center experience and investigated independent predictors of postoperative complications. A retrospective observational analysis was performed on 336 consecutive patients undergoing posterior fossa surgery between January 2015 and January 2025. Positions were classified as sitting (*n* = 250, 74.4%), prone (*n* = 67, 19.9%), and park-bench (*n* = 19, 5.7%). Surgical position, pathological characteristics, operative time, intraoperative venous air embolism (VAE), postoperative complications, hydrocephalus, mortality, and residual lesion were reviewed. Multivariable logistic regression identified predictors of postoperative complications. Overall mortality was 6.8%, with no significant difference between positions (*p* = 0.678). Intraoperative clinically evident VAE occurred in 9 patients (2.7% overall), all in the sitting group (3.6% of sitting cases); Transesophageal Echocardiogram (TEE)/Precordial Doppler detected additional subclinical air embolism events (27 patients in total). Postoperative complications were observed in 33.9% of patients, most commonly cerebrospinal fluid fistula (13.7%) and infection (11.3%), without significant differences between positions (*p* = 0.445). Residual lesions occurred in 16.7% of cases and were less frequent in the sitting position than in the prone position (*p* < 0.001). Operative time was shorter in the sitting position (*p* < 0.001). Hydrocephalus was more common in pediatric patients (*p* < 0.001) and independently predicted postoperative complications (OR=2.06), whereas surgical position did not (*p* > 0.05). In this large single-center cohort, the sitting position provided advantages in operative time and residual lesion rate, while mortality and complication profiles were similar to other positions. The incidence of clinically evident VAE was low and manageable. The findings support that, under meticulous patient selection and standardized anesthesia and neuromonitorization protocols, the sitting position remains a safe and effective option in posterior fossa surgery.

## Introduction

Posterior fossa surgery is one of the most challenging fields in neurosurgery due to the complex and compact anatomy of the brainstem, cerebellum, and cranial nerves [1–4]. In pathologies located in this region, the surgical approach affects not only the extent of resection but also hemodynamic stability, ventilation, and the perioperative complication profile. Therefore, the long-standing debate regarding patient positioning, namely sitting versus horizontal (prone or park-bench), remains relevant based on current evidence [5, 6]. Both historical and contemporary studies show that the choice of position in posterior fossa surgery influences multiple outcomes such as surgical exposure, venous drainage, intracranial pressure, ventilation, and neurophysiological monitoring [2–5].

The sitting position provides the surgeon with a superior operative field, facilitates blood and cerebrospinal fluid (CSF) drainage via gravity, maintains a clean surgical area, enhances anatomical orientation, and allows easier access to midline lesions; however, it has been associated with complications such as venous air embolism (VAE), hemodynamic instability, and pneumocephalus [5–7]. Modern semi-sitting variations aim to minimize these risks through standardized team protocols, including the use of transesophageal echocardiography (TEE), proper central venous catheter (CVC) placement, and head-neck flexion adjustments according to neurophysiological thresholds [7–9]. Classical physiological observations on ventilation and perfusion suggest that the sitting or semi-sitting position may offer thoracic mechanical advantages, yet VAE susceptibility still necessitates careful monitoring. Pediatric series also report that, with appropriate precautions, the sitting position can reduce blood loss and intensive care unit stay [10]; however, VAE cannot be completely eliminated even in children [3].

Horizontal configurations are preferred in many centers because they significantly reduce the risk of VAE and are easier to apply. Nevertheless, they may also be linked with complications such as increased blood loss, limited surgical exposure, macroglossia, pressure sores, brachial plexopathy, visual impairment, and atelectasis [4–6]. Large series comparing sitting and horizontal positions have reported that anesthetic-specific complications (hypotension, end-tidal CO₂ decrease, VAE) are more frequent in the sitting position, whereas hypoxemia and mortality may be higher in the horizontal position; however, functional outcomes do not differ significantly between groups [4, 6]. Evidence increasingly supports that in complex posterior fossa cases, the choice of position depends on lesion location, surgical and anesthetic team experience, and patient characteristics such as ASA score and height [4, 6, 11].

Recent literature indicates that the benefit-risk balance of sitting and horizontal positions varies depending on inter-center differences in practice, case mix, and the availability of monitoring tools such as TEE, capnography, neuromonitorized endotracheal tubes, and somatosensory/motor/brainstem auditory evoked potentials. The advantages in surgical exposure and blood loss are counterbalanced by the risks of VAE and hemodynamic instability, and outcomes remain heterogeneous between pediatric and adult cohorts [1–11]. Moreover, the impact of different pathological diagnoses on position selection and complication risk has not been fully clarified. In this context, the present study aimed to compare the effects of sitting, prone, and park-bench positions on surgical outcomes (operative time, residual lesion, mortality), intraoperative events (VAE), and postoperative complications in patients undergoing posterior fossa surgery using a single-center observational design. Additionally, we aimed to identify independent predictive factors associated with the development of postoperative complications.

## Methods

### Study design

This research was designed as a single-center, retrospective, and observational study. It was conducted by reviewing the medical records of patients who underwent posterior fossa surgery between January 2015 and January 2025. The study was carried out in accordance with the principles of the Declaration of Helsinki and was approved by the Gaziantep University Faculty of Medicine Ethics Committee (Approval No: 2025/392).

### Study population and patient selection

A total of 336 patients who underwent surgical intervention for intracranial lesions located in the posterior fossa region (tumor, hematoma, abscess, etc.) between 2015 and 2025 were included. The inclusion criteria were as follows: (i) patients with complete operative, postoperative follow-up, and pathological data, (ii) those who underwent surgery for an intracranial lesion located in the posterior fossa, and (iii) patients with a confirmed histopathological diagnosis. Patients with missing medical or operative data or insufficient documentation were excluded. All data were retrospectively obtained from the hospital information system and operative notes.

### Surgical procedure

The choice of operative position (sitting versus prone or park-bench) was not randomized and was determined by the attending neurosurgical team based on patient- and lesion-related factors. These included patient age, ASA physical status, and lesion localization. In particular, older patients were more frequently operated in the prone position, whereas the sitting position was preferred in selected pediatric and adult patients when improved venous drainage and surgical visualization were deemed advantageous. All patients underwent standard preoperative anesthetic evaluation, and positioning decisions were made prior to surgery without consideration of intraoperative outcomes.

All surgeries were performed by two experienced neurosurgical teams using retrosigmoid, suboccipital-midline, or far-lateral approaches. Preoperative positioning was selected according to lesion location and patient factors, including ASA score, and age. In the sitting position, patients were fixed with a Mayfield head holder in slight flexion; chest and pelvic straps ensured stability, and elastic bandages or pneumatic compression were applied to the legs. For the prone position, the head was kept in slight flexion and midline, with the eyes and pressure points protected by gel pads. In the park-bench position, patients were placed in lateral decubitus with the head aligned to avoid vertebral artery or jugular vein compression; torso supports and cushions maintained stability, and the upper arm was positioned forward with axillary rolls. Surgical navigation, operating microscope, and intraoperative neuromonitoring were used routinely. Blood loss, operative time, and positioning duration were documented. Standard VAE precautions were applied in all positions, and hemodynamic parameters were continuously monitored by anesthesia throughout the procedure.

### Anesthesiological management

All patients received balanced anesthesia with propofol, fentanyl or sufentanil, and rocuronium or atracurium; intubation used a flexometallic tube. Monitoring included a 5-lead ECG, invasive arterial pressure, EtCO₂, SpO₂, urine output, and temperature. In the sitting position, additional precautions were taken: a central venous catheter was placed at the right atrial level for air aspiration, and VAE surveillance was performed with TEE or precordial Doppler. Pneumatic compression or anti-shock trousers were used on the legs. A > 5 mmHg EtCO₂ drop, > 15% heart-rate increase, or > 20% systolic pressure decrease was considered “high-probability air embolism.” In the park-bench position, anesthetic management was similar, though VAE risk was lower and TEE was replaced by standard monitoring. Hemodynamic stability was ensured before positioning, and normothermia maintained with active warming systems. After surgery, all patients were transferred to neurosurgical intensive care for hemodynamic stabilization, neurological assessment, and routine CT imaging.

### Data collection and variables

Demographic data (age, sex), lesion characteristics (size, histopathological diagnosis), surgical parameters (patient position during surgery, operative duration, intraoperative complications), and postoperative outcomes (hydrocephalus, mortality, postoperative complications, presence of residual lesion) were included in the analysis. Lesion size was calculated by multiplying the largest axial and sagittal diameters, and this value was used in all analyses. Lesion types were classified according to pathology reports under 14 diagnostic categories. To ensure statistical robustness, lesions with four or fewer cases were grouped under the “Other” category. Surgical positions were evaluated in three groups: sitting, prone, and park-bench. Complication types were recorded separately as intraoperative and postoperative.

### Statistical analysis

Data were analyzed using SPSS 25.0 (IBM Corp., Armonk, NY). Categorical variables were presented as number (%) and continuous variables as mean ± SD or median (Q1–Q3), depending on distribution. Normality was tested with Shapiro–Wilk and Kolmogorov–Smirnov, and variance homogeneity with Levene’s test. Between-group comparisons used Student’s t-test for normally distributed variables and Mann–Whitney U for nonparametric data. For comparisons among three groups, Kruskal–Wallis was applied, followed by Dunn-Bonferroni post-hoc tests when significant. Categorical variables were compared using Pearson’s chi-square or Fisher’s exact test based on expected counts. Correlations between numerical variables were analyzed using Spearman’s method. Risk factors for postoperative complications were first assessed with univariable logistic regression, and significant or clinically relevant variables were entered into a multivariable logistic regression model. Odds ratios (OR) with 95% confidence intervals (CI) were reported. A p-value < 0.05 was considered statistically significant.

## Results

A total of 336 patients who underwent posterior fossa surgery were included. The mean age was 26.8 ± 22.86 years (0–79), mean lesion size 12.24 ± 7.1 cm² (0.5–36), and mean operative time 268.61 ± 32.96 min (145–355). Of all patients, 49.4% (*n* = 166) were female and 50.6% (*n* = 170) male. The most common pathologies were medulloblastoma (25.9%, *n* = 87) and pilocytic astrocytoma (19.3%, *n* = 65), followed by metastasis (13.7%, *n* = 46), ependymoma (6.8%, *n* = 23), meningioma (6.3%, *n* = 21), hemangioblastoma (4.2%, *n* = 14), hematoma (3.9%, *n* = 13), epidermoid cyst (3.6%, *n* = 12), cavernoma (2.4%, *n* = 8), abscess (2.1%, *n* = 7), arachnoid cyst (2.1%, *n* = 7), dermoid cyst (2.1%, *n* = 7), and choroid plexus papilloma (2.1%, *n* = 7). 19 cases (5.7%) with ≤ 4 occurrences were grouped as “others,” including glioblastoma (1.2%, *n* = 4), lymphoma (1.2%, *n* = 4), arteriovenous malformation (0.9%, *n* = 3), schwannoma (0.9%, *n* = 3), reactive gliosis (0.6%, *n* = 2), atypical teratoid rhabdoid tumor (0.3%, *n* = 1), neurocytoma (0.3%, *n* = 1), and teratoma (0.3%, *n* = 1).

Regarding surgical position, 74.4% (*n* = 250) of patients were operated in the sitting, 19.9% (*n* = 67) in the prone, and 5.7% (*n* = 19) in the park-bench position. Mortality from the intraoperative period up to 30 days after surgery was 6.8% (*n* = 23). Intraoperative complications occurred in 2.7% of patients (*n* = 9), all of which were clinically evident air embolisms; although TEE detected a total of 27 cases of air embolism, only those 9 cases with clinical manifestations were classified as intraoperative complications. Postoperative complications developed in 33.9% (*n* = 114), most commonly CSF fistula (13.7%, *n* = 46) and infection (11.3%, *n* = 38); three patients (0.9%) had both, and one (0.3%) had CSF fistula with mortality. Rare complications included epidural hematoma (0.3%, *n* = 1), facial palsy (0.3%, *n* = 1), pneumothorax (0.3%, *n* = 1), isolated postoperative mortality (6.3%, *n* = 21), and fifth–eighth cranial nerve palsy (0.3%, *n* = 1). Postoperative hydrocephalus occurred in 18.2% (*n* = 61). Residual lesion was seen in 16.7% (*n* = 56), while gross total resection was achieved in 83.3% (*n* = 280).

**Fig. 1:**
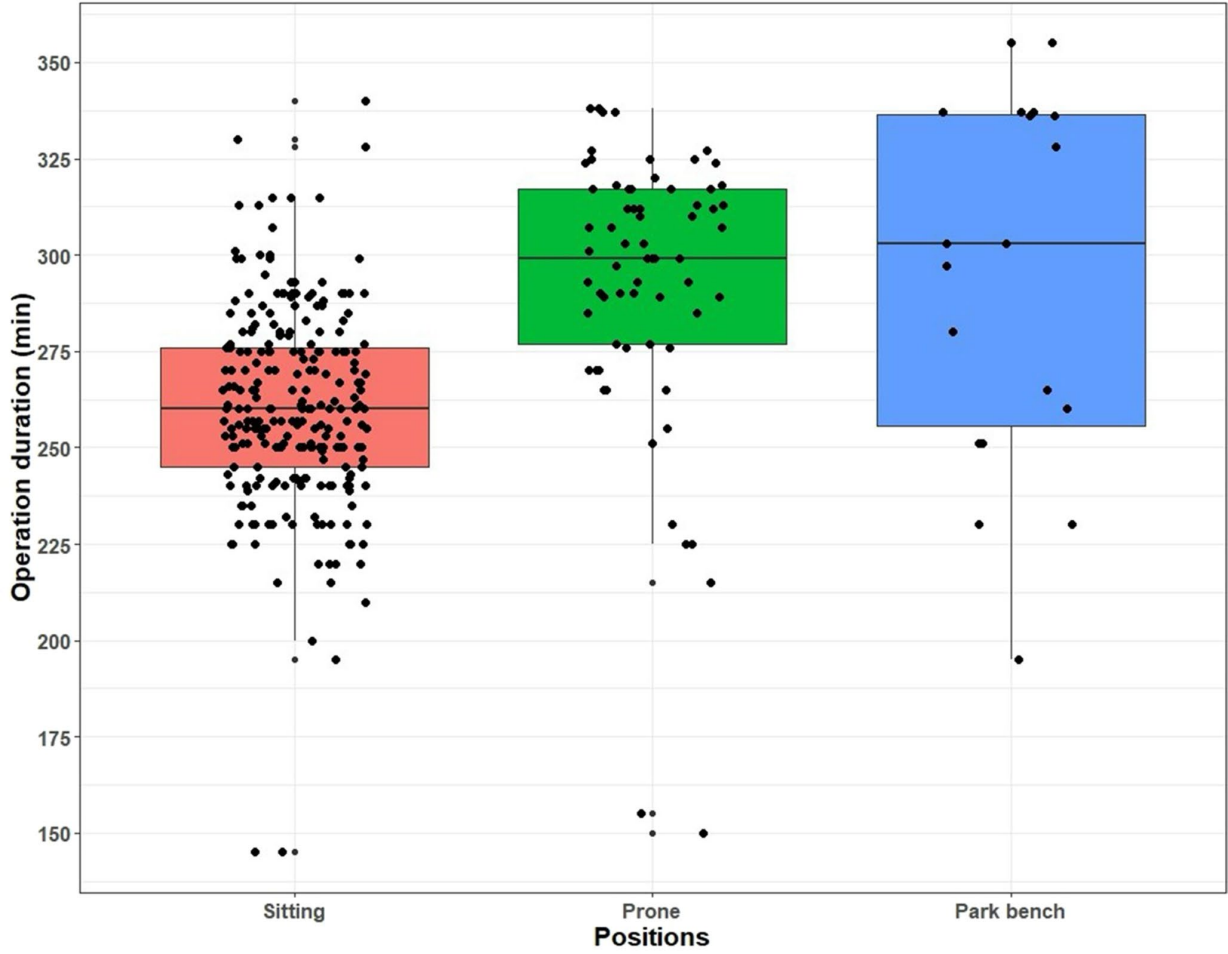
Boxplots with jitter showing the distribution of operation times among surgical positions

When compared by surgical position, mortality, hydrocephalus, and intraoperative and postoperative complication rates did not differ significantly (*p* > 0.05, Table 1). Mortality was 7.2% in the sitting, 7.5% in the prone, and 0% in the park-bench position, while hydrocephalus occurred in 19.6%, 17.9%, and 0% of patients, respectively. Intraoperative complications were seen only in the sitting group (3.6%), and postoperative complications occurred in 35.2%, 32.8%, and 21.1% of the sitting, prone, and park-bench groups, respectively. Residual lesion rates differed significantly among positions (*p* < 0.001); post-hoc analysis showed significance between sitting and prone (*p* < 0.001), but not between sitting and park-bench (*p* = 0.774) or prone and park-bench (*p* = 0.056). The proportion of female patients was 50.8% in the sitting, 38.8% in the prone, and 68.4% in the park-bench group (*p* = 0.051). When key determinants of group allocation were evaluated, significant differences were observed among groups in terms of ASA classification as well as Location–Position 1 and Location–Position 2 distributions (all *p* < 0.001, Table 1). Age differed significantly among positions (*p* < 0.001), with park-bench patients being older than both sitting (*p* < 0.001) and prone (*p* = 0.026) groups, and prone patients older than sitting (*p* = 0.007). Lesion size was similar across groups (*p* = 0.382; median 12 cm²). Operative time differed significantly (*p* < 0.001), being shorter in the sitting group compared with the prone (*p* < 0.001) and park-bench (*p* = 0.001) groups; prone and park-bench times were similar (*p* = 1.000). Median operative times were 260 min (244.5–276.3) in the sitting, 299 min (277–317) in the prone, and 303 min (251–337) in the park-bench group (Fig. 1).

**Table 1:** Comparison of demographic variables, mortality, complications, and surgical outcomes according to surgical position

			Positions		*P* values	Post-hoc *P* values
		Sitting (*n* = 250)	Prone (*n* = 67)	Park bench (*n* = 19)		
Mortality	**No**	232 (92.8%)	62 (92.5%)	19 (100%)	0.678^b^	-
	**Yes**	18 (7.2%)	5 (7.5%)	0 (0%)		
Hydrocephalus	**No**	201 (80.4%)	55 (82.1%)	19 (100%)	0.076^b^	-
	**Yes**	49 (19.6%)	12 (17.9%)	0 (0%)		
Intra operative complication	**No**	241 (96.4%)	67 (100%)	19 (100%)	0.333^b^	-
	**Yes**	9 (3.6%)	0 (0%)	0 (0%)		
Post operative complication	**No**	162 (64.8%)	45 (67.2%)	15 (78.9%)	0.445^a^	-
	**Yes**	88 (35.2%)	22 (32.8%)	4 (21.1%)		
Residual lesion	**No**	218 (87.2%)	45 (67.2%)	17 (89.5%)	**< 0.001** ^**a**^	**1–2: <0.001**
	**Yes**	32 (12.8%)	22 (32.8%)	2 (10.5%)		1–3: 0.774
						2–3: 0.056
Gender	**F**	127 (50.8%)	26 (38.8%)	13 (68.4%)	0.051^a^	-
	**M**	123 (49.2%)	41 (61.2%)	6 (31.6%)		
ASA	**I**	47 (18.8%)	11 (16.4%)	6 (31.6%)	**< 0.001** ^**b**^	-
	**II**	182 (72.8%)	20 (29.9%)	9 (47.4%)		
	**III**	21 (8.4%)	33 (49.3%)	4 (21.1%)		
	**IV**	0 (0%)	3 (4.5%)	0 (0%)		
Location-Position–1	**Deep-seated**	207 (82.8%)	8 (11.9%)	12 (63.2%)	**< 0.001** ^**a**^	-
	**Superficial**	43 (17.2%)	59 (88.1%)	7 (36.8%)		
Location-Position–2	**Near the tentorium (Superior)**	184 (73.6%)	21 (31.3%)	0 (0%)	**< 0.001** ^**b**^	-
	**Near the foramen magnum (Inferior)**	66 (26.4%)	46 (68.7%)	0 (0%)		
	**Lateral location**	0 (0%)	0 (0%)	19 (100%)		
Age		15 (6–37)	29 (8–61)	55 (44–60)	**< 0.001** ^**c**^	**1–2: 0.007** **1–3: <0.001** **2–3: 0.026**
Lesion size (cm^2^)		12 (6.2–18)	12 (6–16)	12 (6–12)	0.382^c^	
Operation duration (min)		260 (244.5–276.3)	299 (277–317)	303 (251–337)	**< 0.001** ^**c**^	**1–2: <0.001** **1–3: 0.001** 2–3: 1.000

^a^Chi-square test with n (%)

^b^Fisher exact test with n (%)

^c^Kruskal Wallis test with median (Q1, Q3)

*F*: Female, *M* : Male

There were no statistically significant differences in mortality, postoperative complication, or residual lesion rates among different pathology types (*p* = 0.568, *p* = 0.417, and *p* = 0.699, respectively). The highest mortality was observed in the “other” lesion group (21.1%), followed by choroid plexus papilloma (14.3%), ependymoma (13.0%), and medulloblastoma (8.0%) (Table 2). No mortality occurred in cases of meningioma, hematoma, epidermoid cyst, cavernoma, abscess, arachnoid cyst, or dermoid cyst. The highest postoperative complication rate was found in the “other” group (52.6%), followed by meningioma (47.6%), epidermoid cyst (41.7%), medulloblastoma (40.2%), ependymoma (34.8%), hematoma (30.8%), and pilocytic astrocytoma (30.8%). The highest residual lesion rates were observed in cavernomas (25.0%) and medulloblastomas (24.1%) (Table 2).

**Table 2:** Comparison of mortality, postoperative complications, and residual rates according to pathology types

Pathology	Mortality		Postoperative complication		Residual lesion	
	No (*n* = 313)	Yes (*n* = 23)	No (*n* = 222)	Yes (*n* = 114)	No (*n* = 280)	Yes (*n* = 56)
Medulloblastoma	80 (92.0%)	7 (8.0%)	52 (59.8%)	35 (40.2%)	66 (75.9%)	21 (24.1%)
Pilocytic astrocytoma	61 (93.8%)	4 (6.2%)	45 (69.2%)	20 (30.8%)	55 (84.6%)	10 (15.4%)
Metastasis	43 (93.5%)	3 (6.5%)	34 (73.9%)	12 (26.1%)	37 (80.4%)	9 (19.6%)
Ependymoma	20 (87.0%)	3 (13.0%)	15 (65.2%)	8 (34.8%)	21 (91.3%)	2 (8.7%)
Meningioma	21 (100.0%)	0 (0.0%)	11 (52.4%)	10 (47.6%)	20 (95.2%)	1 (4.8%)
Hemangioblastoma	13 (92.9%)	1 (7.1%)	11 (78.6%)	3 (21.4%)	13 (92.9%)	1 (7.1%)
Hematoma	13 (100.0%)	0 (0.0%)	9 (69.2%)	4 (30.8%)	11 (84.6%)	2 (15.4%)
Epidermoid cyst	12 (100.0%)	0 (0.0%)	7 (58.3%)	5 (41.7%)	11 (91.7%)	1 (8.3%)
Cavernoma	8 (100.0%)	0 (0.0%)	7 (87.5%)	1 (12.5%)	6 (75.0%)	2 (25.0%)
Abscess	7 (100.0%)	0 (0.0%)	5 (71.4%)	2 (28.6%)	6 (85.7%)	1 (14.3%)
Arachnoid cyst	7 (100.0%)	0 (0.0%)	5 (71.4%)	2 (28.6%)	6 (85.7%)	1 (14.3%)
Dermoid cyst	7 (100.0%)	0 (0.0%)	6 (85.7%)	1 (14.3%)	7 (100.0%)	0 (0.0%)
Choroid plexus papilloma	6 (85.7%)	1 (14.3%)	6 (85.7%)	1 (14.3%)	6 (85.7%)	1 (14.3%)
Others	15 (78.9%)	4 (21.1%)	9 (47.4%)	10 (52.6%)	15 (78.9%)	4 (21.1%)

**Fig. 2:**
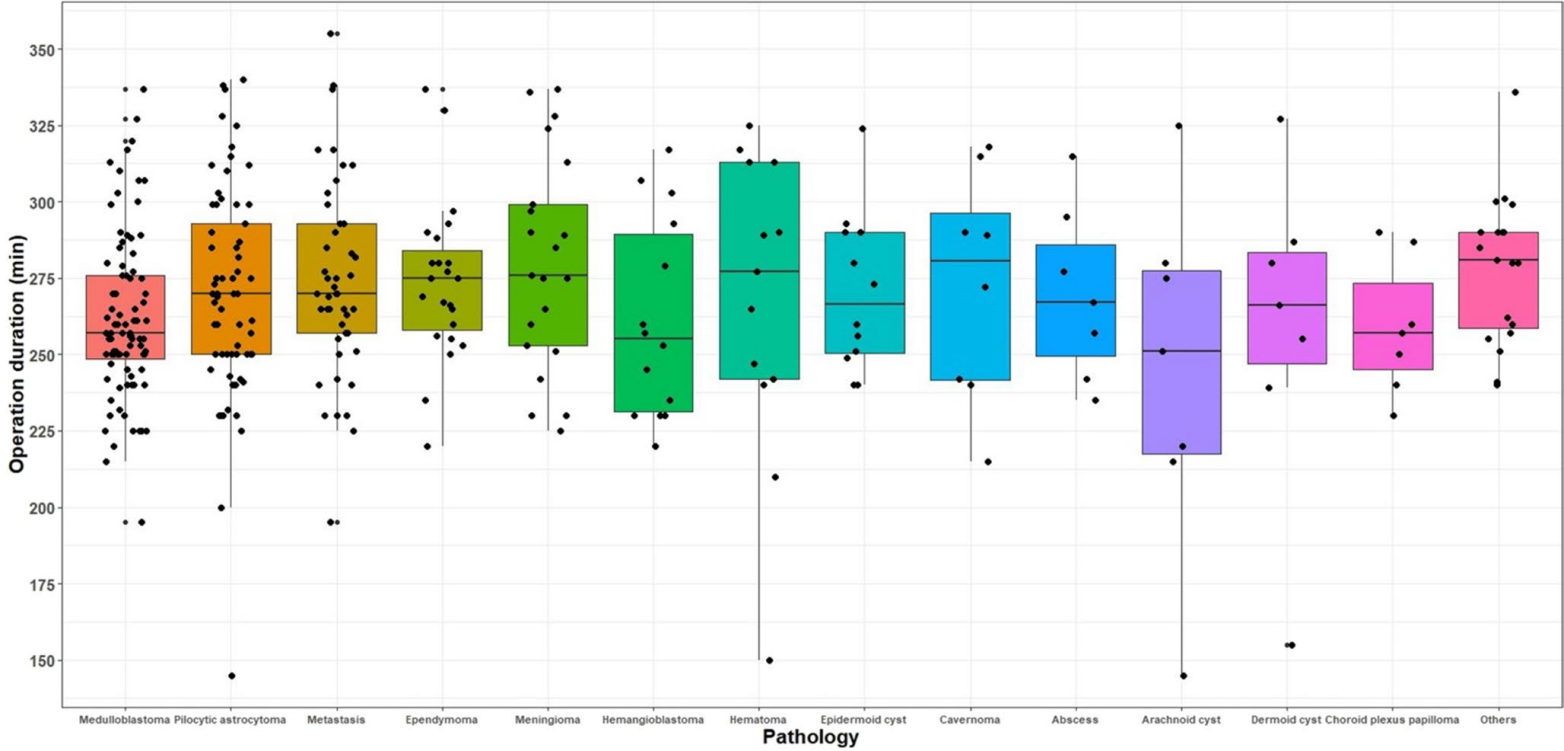
Boxplots with jitter showing the distribution of operation times among pathology groups

No significant difference was found among pathology groups in terms of operative time (*p* = 0.227, Fig. 2). A weak but statistically significant positive correlation was found between lesion size and operative time (*r* = 0.161, *p* = 0.003).

There were no statistically significant associations between operative time and intraoperative complication, postoperative complication, or residual lesion presence (*p* > 0.05, Table 3). The median lesion size was 12 cm² (6–16) in patients without intraoperative complications and 13.75 cm² (9.75–21.25) in those with complications. The mean lesion size was 11.96 ± 6.68 cm² in patients without postoperative complications and 12.78 ± 7.86 cm² in those with complications. Similarly, the mean lesion size was 12.17 ± 7.13 cm² in patients without residual lesions and 12.59 ± 6.99 cm² in those with residual lesions.

**Table 3:** Relationship between operation time, intraoperative and postoperative complications, and presence of residual lesion

		n	Operation duration	*P* values
Intraoperative complication	**No**	327	12 (6–16)	0.182^b^
	**Yes**	9	13.75 (9.75–21.25)	
Postoperative complication	**No**	222	11.96 ± 6.68	0.340^a^
	**Yes**	114	12.78 ± 7.86	
Residual lesion	**No**	280	12.17 ± 7.13	0.683^a^
	**Yes**	56	12.59 ± 6.99	

^a^Student’s t-test with mean ± standard deviation

^b^Mann Whitney U test with median (Q1, Q3)

A statistically significant difference was found between hydrocephalus and postoperative complication rates (30.5% in patients without hydrocephalus, 49.2% in those with hydrocephalus; *p* = 0.005, Table 4). However, residual lesion rates were similar (15.3% without hydrocephalus, 23.0% with hydrocephalus; *p* = 0.145).

**Table 4:** Comparison of postoperative complication and residual rates according to the presence of hydrocephalus

		Hydrocephalus		*P* values
		No (*n* = 275)	Yes (*n* = 61)	
Postoperative complication	**No**	191 (69.5%)	31 (50.8%)	**0.005** ^**a**^
	**Yes**	84 (30.5%)	30 (49.2%)	
Residual lesion	**No**	233 (84.7%)	47 (77%)	0.145^a^
	**Yes**	42 (15.3%)	14 (23%)	

^a^Chi-square test with n (%)

No significant difference was found in mortality rates between age groups (*p* = 0.694, Table 5). The mortality rate was 7.4% in patients under 18 years and 6.3% in those aged 18 years or older. The incidence of hydrocephalus differed significantly between age groups (*p* < 0.001), being 26.5% in patients under 18 years and 10.3% in adults. Intraoperative complication rates were similar between groups (2.5% vs. 2.9%; *p* = 1.000). The postoperative complication rate was 38.9% in patients under 18 years and 29.3% in those aged 18 years or older, with no statistically significant difference (*p* = 0.064). Similarly, there was no significant difference in residual lesion rates between age groups (*p* = 0.241); 19.1% in patients under 18 years and 14.4% in adults. Pathological distribution differed significantly between age groups (*p* < 0.001, Table 5). The most common lesion type in patients under 18 years was medulloblastoma (41.4%), followed by pilocytic astrocytoma (32.7%) and ependymoma (9.3%), while in adults, metastasis (26.4%) was most common, followed by meningioma (11.5%), medulloblastoma (11.5%), and hemangioblastoma (8.0%).

**Table 5:** Comparison of mortality, complications, residual rates, and pathological distributions between pediatric and adult age groups

		Age groups		*P* values
		< 18 (n=)	≥ 18 (n=)	
Mortality	**No**	150 (92.6%)	163 (93.7%)	0.694^a^
	**Yes**	12 (7.4%)	11 (6.3%)	
Hydrocephalus	**No**	119 (73.5%)	156 (89.7%)	**< 0.001** ^**a**^
	**Yes**	43 (26.5%)	18 (10.3%)	
Intraoperative complication	**No**	158 (97.5%)	169 (97.1%)	1.000^b^
	**Yes**	4 (2.5%)	5 (2.9%)	
Postoperative complication	**No**	99 (61.1%)	123 (70.7%)	0.064^a^
	**Yes**	63 (38.9%)	51 (29.3%)	
Residual lesion	**No**	131 (80.9%)	149 (85.6%)	0.241^a^
	**Yes**	31 (19.1%)	25 (14.4%)	
Pathology	**Medulloblastoma**	67 (41.4%)	20 (11.5%)	**< 0.001** ^**b**^
	**Pilocytic astrocytoma**	53 (32.7%)	12 (6.9%)	
	**Metastasis**	0 (0.0%)	46 (26.4%)	
	**Ependymoma**	15 (9.3%)	8 (4.6%)	
	**Meningioma**	1 (0.6%)	20 (11.5%)	
	**Hemangioblastoma**	0 (0.0%)	14 (8.0%)	
	**Hematoma**	2 (1.2%)	11 (6.3%)	
	**Epidermoid cyst**	2 (1.2%)	10 (5.7%)	
	**Cavernoma**	4 (2.5%)	4 (2.3%)	
	**Abscess**	2 (1.2%)	5 (2.9%)	
	**Arachnoid cyst**	4 (2.5%)	3 (1.7%)	
	**Dermoid cyst**	5 (3.1%)	2 (1.1%)	
	**Choroid plexus papilloma**	1 (0.6%)	6 (3.4%)	
	**Others**	6 (3.7%)	13 (7.5%)	

^a^Chi-square test with n (%)

^b^Fisher exact test with n (%)

In univariable logistic regression analysis, lesion size (*p* = 0.314), operative time (*p* = 0.454), intraoperative complication (*p* = 0.970), and surgical position (sitting vs. park-bench: *p* = 0.218; prone vs. park-bench: *p* = 0.328) showed no significant effect on postoperative complications (Table 6). Residual lesion (No vs. Yes) approached significance (*p* = 0.067; OR = 1.87). In contrast, sex and the presence of hydrocephalus were significantly associated with postoperative complication development. Female sex was associated with a 1.68-fold higher risk compared to males (*p* = 0.026; OR = 1.68), and the presence of hydrocephalus increased the risk 2.2-fold compared to those without hydrocephalus (*p* = 0.006; OR = 2.2). Variables significant at *p* < 0.10 in the univariable model (residual lesion, sex, and hydrocephalus) were included in the multivariable model. Although age was not significant in the univariable model (*p* = 0.176), it was included to control for potential confounding. In the multivariable model, the effect of residual lesion was not significant (*p* = 0.053) and was excluded from the final model. In the final model, sex (*p* = 0.027; OR = 1.68) and hydrocephalus (*p* = 0.015; OR = 2.06) remained independent predictors (Table 6). Accordingly, the presence of hydrocephalus approximately doubled the likelihood of postoperative complications, while female sex increased the risk by about 1.7 times.

**Table 6:** Findings of univariable and multivariable logistic regression analyses regarding the predictive role of risk factors on postoperative complications

	Univariable Model		Final Multivariable Model	
	*P* values	OR (CI 95%)	*P* values	OR (CI 95%)
Lesion size	0.314	-	-	-
Operation duration	0.454	-	-	-
Intraoperative complication (Yes vs. No)	0.970	-	-	-
Positions (Sitting vs. Park beach)	0.218	-	-	-
Positions (Prone vs. Park beach)	0.328	-	-	-
Age	0.176	-	0.370	-
Residual lesion (No vs. Yes)	**0.067**	1.87 (0.96 − 3.64)	**-**	-
Gender (Female vs. male)	**0.026**	**1.68 (1.06–2.65)**	**0.027**	**1.68 (1.06–2.67)**
Hydrocephalus (Yes vs. No)	**0.006**	**2.2 (1.25–3.87)**	**0.015**	**2.06 (1.15–3.69)**
Multivariate model: Nagelkerke R Square: 0.053				

Values below *P* < 0.05 were shown bold

*OR,* Odds ratio; *CI,* Confidence interval

## Discussion

Although the safety and efficacy of the sitting position in posterior fossa surgery has long been debated, recent comparative studies more clearly demonstrate its advantages under modern monitoring conditions. In the pioneering series of Black et al. (1988) with 579 cases, the VAE rate reached 45%, yet no VAE-related mortality occurred [12], indicating that VAE is now largely preventable and manageable with advanced monitoring. The same study reported reduced blood loss and transfusion needs in the sitting versus horizontal position (359 mL vs. 507 mL) [12]. Rath et al. (2007) similarly found less blood loss (320 vs. 638 mL, *p* = 0.003), shorter operative time (*p* = 0.037), and higher lower cranial nerve preservation (76% vs. 52%, *p* < 0.05) in 260 cases [13]. Considered together, these two classic studies suggest that the sitting position is a safe alternative that increases both surgical efficiency and rates of neurological preservation with appropriate patient selection, and that concerns about early complications have largely been alleviated today thanks to advanced neuroanesthesia support.

More recent studies by Spektor et al. (2015), Mavarez-Martinez et al. (2020), and Radu et al. (2024) also found no significant differences between positions in mortality, postoperative complications, or functional recovery [4–6]. Spektor et al. noted better facial nerve preservation in the lateral position (91% vs. 70%), with similar resection and complication rates [4]. Mavarez-Martinez et al. reported higher VAE incidence in the sitting position (40%) without major morbidity, while blood loss and transfusion requirements were higher in horizontal positions [5]. Radu et al. observed more anesthetic complications and hypotension in the sitting position but higher mortality in the park-bench group (6.0%) [6]. The common conclusion of these studies is that although the sitting position is more susceptible to intraoperative hemodynamic fluctuations, it offers clear advantages in terms of surgical visualization, hemostasis, and resection success when managed by experienced teams. In our series as well, the sitting position supports these findings with a significantly shorter operative time (260 min versus 299–303 min), a significantly lower residual rate (%12.8 versus %32.8), and similar complication rates.

Additionally, Khalaveh et al. (2024) emphasized the importance of individual risk factors for VAE development in a series of 202 semi-sitting cases, reporting that patient height (*p* = 0.04) and ASA class (*p* = 0.03) were significant determinants. The higher incidence of VAE in taller patients and those with lower ASA class highlights the necessity of risk-based patient selection in the sitting position [11]. In this respect, careful hemodynamic monitoring and ensuring optimal venous return in our study also kept complications at a minimal level. In light of all these data, it can be stated that although the sitting position involves physiological risks such as VAE and hypotension, these are controllable with modern neuroanesthesia, neuromonitoring, and meticulous patient selection, while it remains a superior option in terms of surgical visibility, reduced blood loss, and resection efficacy.

In the randomized clinical study by Dilmen et al. (2023), the effects of the sitting and prone positions used in posterior fossa surgeries on cerebral oxygenation were compared [14]. Measurements with near-infrared spectroscopy (NIRS) showed that cerebral oxygenation slightly decreased after positioning in both groups, but there was no significant difference between the groups. Although mean arterial pressure (MAP) and heart rate were notably lower in the sitting position, no clinically significant desaturation developed. The authors suggested that if the lower limit of MAP is maintained at a higher level, the sitting position could demonstrate an enhancing effect on cerebral oxygenation [14]. In our series, however, findings regarding hemodynamic parameters (MAP, heart rate, rSO₂) could not be reported.

Hernández-Palazón et al. (2003) evaluated the effect of different anesthesia techniques on postoperative pneumocephalus in 90 posterior fossa surgeries performed in the sitting position and found varying degrees of pneumocephalus in all patients, with symptoms in only two cases [15]. Similarly, Sloan (2010) reported a supratentorial pneumocephalus (STP) incidence of 42.1% in 95 cases, noting that air volume ranged between 6 and 280 cm³ and that most patients showed no clinical deterioration [16]. Hong et al. (2015) demonstrated that normobaric hyperoxia accelerated air resorption, while Sachkova et al. (2018) identified a 3.9% rate of ventricular tension pneumocephalus and highlighted opening of the fourth ventricle as the strongest risk factor [17, 18]. In our series, postoperative pneumocephalus was common but largely asymptomatic; only three patients developed tension pneumocephalus requiring intervention. Overall, these findings indicate that pneumocephalus occurs more frequently in the sitting position than in horizontal configurations but is generally self-limiting and manageable through careful irrigation, pressure control, hyperoxia, or conservative approaches with close monitoring. Consistent with previous literature, our results show that the sitting position maintains a manageable pneumocephalus risk profile when supported by modern neuroanesthesia.

In pediatric populations, the safety and efficacy of the sitting position are supported by both clinical outcomes and standardized anesthetic principles. Orliaguet et al. (2001) reported lower transfusion needs, fewer intraoperative complications, and shorter intubation, ICU, and hospital stays without increased morbidity [10]. Baro et al. (2019) similarly found no significant differences between sitting and prone groups in low-grade tumors—particularly pilocytic astrocytoma—and reported only one clinically meaningful VAE [19]. In our pediatric subgroup, mortality and overall complication rates were comparable across positions, while operative time was shorter and residual lesions were less frequent in the sitting position. These findings align with pediatric literature and support the efficiency advantage of the sitting position when appropriately selected.

From a technical and anesthetic perspective, Klein et al. (2021) emphasized that both the sitting and prone configurations can be safely implemented without opposing each other, through standardized steps such as the telovelar approach, management of hemodynamic fluctuations, careful dural closure, and preservation of the vermis and dentate [20]. This approach is consistent with the findings of Hermann et al. (2023) in their semi-sitting series under four years of age, in which VAE was mostly low grade and manageable, no major cardiopulmonary events or mortality were reported, and postoperative pneumocephalus was common yet asymptomatic [21]. In our series, intraoperative VAE in the sitting group was infrequent, and the majority of cases were detected without clinical symptoms; the advantage in operative time, together with equivalence in complications, suggests that the sitting position can be safely maintained in pediatric patients when performed by an experienced team with invasive monitoring and protocol-based standardization.

Krauss et al. (2025) examined postoperative functional outcomes by comparing sitting and nonsitting positions in posterior fossa metastasis surgery [8]. In their retrospective two-center analysis, one center used only the sitting position while the other used only the nonsitting position. The findings showed that the Karnofsky performance score (KPS) was significantly lower in the sitting group, with a marked increase in the proportion of patients falling below 60%. Based on these results, the authors suggested that the sitting position may be associated with less favorable functional outcomes in metastatic cases and recommended the nonsitting position. However, the generalizability of these findings is limited by the exclusive focus on metastases and the lack of full standardization between centers regarding patient selection, lesion characteristics, and surgical complexity [8]. In our series, the sitting position was more advantageous regarding operative time, and no significant differences were observed in mortality or postoperative complications. Thus, unlike the metastasis-specific results of Krauss et al. (2025), our broader cohort demonstrated a neutral safety and functional profile for the sitting position across diverse pathologies.

With technological advances, the standardization of the semi-sitting position and its navigation-assisted use have significantly improved safety in modern neurosurgery. Hermann et al. (2015) showed that target accuracy using an electromagnetic (EM) navigation system remained essentially unchanged between the supine (2.48 mm) and semi-sitting (2.5 mm) positions, indicating that positioning did not affect accuracy [22]. The elimination of the “line-of-sight” limitation with EM navigation also facilitated venous sinus preservation and uninterrupted workflow [22]. These findings align with the principles of the approximately one-hundred-step optimized semi-sitting protocol described by Roman et al. (2023), which emphasizes multidisciplinary coordination, integrated anesthesia and neuromonitoring, and rigorous hemodynamic management rather than solely individual surgical skill [7]. In our series, the significantly shorter operative time in the sitting position and comparable complication rates likewise reflect the benefits of this modern standardization and technology integration. Collectively, the studies of Hermann and Roman indicate that the semi-sitting position should be considered a structured, technology-supported safety system—applicable in both adult and pediatric patients when performed by experienced teams with meticulous monitoring [22, 7]. Our results also demonstrate that this contemporary safety approach can be effectively incorporated into sitting-position surgery.

### Strengths and limitations

The strengths of this study include its large sample size (*n* = 336), use of standardized surgical and neuromonitoring protocols and detailed anesthesia procedures within a single center, and advanced statistical comparisons across three surgical positions, which together reduced inter-center heterogeneity and enhanced internal validity. However, the retrospective design and position selection based on clinical requirements and surgeon preference may have introduced selection bias and unmeasured confounding; although major clinical determinants were adjusted for using multivariable modeling, causal inferences cannot be drawn. The wide pathological spectrum and the use of lesion area (product of two diameters) rather than true volume may also have introduced methodological variability, although this approach was applied consistently across groups and accompanied by pathology-specific subgroup analyses. In addition, the absence of systematic long-term functional follow-up (e.g., KPS and cranial nerve outcomes) across the cohort may limit external validity, as the study primarily focused on surgical and early postoperative safety indicators; therefore, these findings should be confirmed in prospective, function-oriented studies with balanced group designs.

## Conclusions

In this single-center, large, and heterogeneous cohort, the sitting position demonstrated superiority in surgical efficiency in posterior fossa surgery, characterized by a significantly shorter operative time and a lower residual lesion rate compared with the prone position. In contrast, mortality, intraoperative and postoperative complication rates, and the incidence of hydrocephalus did not differ among positions. Intraoperative VAE occurred only in the sitting group at a low rate, with the majority of cases being clinically silent, and was clinically manageable. In multivariable analysis, hydrocephalus was identified as a significant risk factor for postoperative complications, whereas surgical position was not. In conclusion, with meticulous patient selection, advanced monitoring, and standardized anesthesia protocols, the sitting position represents a safe option in posterior fossa surgery and holds the potential to enhance surgical efficiency; optimal outcomes can be achieved through careful patient selection and perioperative risk management.

## Data Availability

No datasets were generated or analysed during the current study.
